# Silver Citrate

**Published:** 1898-09

**Authors:** 


### Silver Citrate.

This is recommended by Prof. Allsnell as an antiseptic and bactericidal application to wounds. It may be used pure, or incorporated in ointment or water solution. As a powder it may be applied directly to wounds or granulating surfaces of the skin or mucous membranes. It may be thinly dusted over them once, or at intervals of several days, according to indications.—*Practical Druggist.*

A Boston paper says that "a message cast into the sea in midocean by a New York man in a bottle has been picked up near France." But what became of the New York man in a bottle?—*Chicago Times-Herald.*

Perhaps he went down in the wreck with the English language.

